# Identification of antennal alternative splicing by combining genome and full-length transcriptome analysis in *Bactrocera dorsalis*

**DOI:** 10.3389/fphys.2024.1384426

**Published:** 2024-06-17

**Authors:** Qi Wang, Jie Zhang, Chenhao Liu, Chuanjian Ru, Qian Qian, Minghuan Yang, Shanchun Yan, Wei Liu, Guirong Wang

**Affiliations:** ^1^ Key Laboratory of Sustainable Forest Ecosystem Management-Ministry of Education, Northeast Forestry University, Harbin, China; ^2^ Shenzhen Branch, Guangdong Laboratory of Lingnan Modern Agriculture, Genome Analysis Laboratory of the Ministry of Agriculture and Rural Affairs, Agricultural Genomics Institute at Shenzhen, Chinese Academy of Agricultural Sciences, Shenzhen, China

**Keywords:** Bactrocera dorsalis, antennae, full-length transcripts, sex-biased differential alternative splicing, isoform switch

## Abstract

Alternative splicing is an essential post-transcriptional regulatory mechanism that diversifies gene function by generating multiple protein isoforms from a single gene and act as a crucial role in insect environmental adaptation. Olfaction, a key sense for insect adaptation, relies heavily on the antennae, which are the primary olfactory organs expressing most of the olfactory genes. Despite the extensive annotation of olfactory genes within insect antennal tissues facilitated by high-throughput sequencing technology advancements, systematic analyses of alternative splicing are still relatively less. In this study, we focused on the oriental fruit fly (*Bactrocera dorsalis*), a significant pest of fruit crops. We performed a detailed analysis of alternative splicing in its antennae by utilizing the full-length transcriptome of its antennal tissue and the insect’s genome. The results revealed 8600 non-redundant full-length transcripts identified in the oriental fruit fly antennal full-length transcriptome, spanning 4,145 gene loci. Over 40% of these loci exhibited multiple isoforms. Among these, 161 genes showed sex-biased isoform switching, involving seven different types of alternative splicing. Notably, events involving alternative transcription start sites (ATSS) and alternative transcription termination sites (ATTS) were the most common. Of all the genes undergoing ATSS and ATTS alternative splicing between male and female, 32 genes were alternatively spliced in protein coding regions, potentially affecting protein function. These genes were categorized based on the length of the sex-biased isoforms, with the highest difference in isoform fraction (dIF) associated with the ATSS type, including genes such as *BdorABCA13*, *BdorCAT2*, and *BdorTSN3.* Additionally, transcription factor binding sites for doublesex were identified upstream of both BdorABCA13 and BdorCAT2. Besides being expressed in the antennal tissues, *BdorABCA13* and *BdorCAT2* are also expressed in the mouthparts, legs, and genitalia of both female and male adults, suggesting their functional diversity. This study reveals alternative splicing events in the antennae of *Bactrophora dorsalis* from two aspects: odorant receptor genes and other types of genes expressed in the antennae. This study not only provides a research foundation for understanding the regulation of gene function by alternative splicing in the oriental fruit fly but also offers new insights for utilizing olfaction-based behavioral manipulation techniques to manage this pest.

## 1 Introduction

Alternative splicing (AS) is an essential post-transcriptional regulatory mechanism and enables the generation of structurally variable transcripts from a single gene (Verta and Jacobs, 2022). This mechanism is widespread in insects, greatly enriching the diversity of insect mRNAs and proteins. For instance, in the *Drosophila melanogaster* genome, the proportion of alternative splicing events is as high as 40% (Stolc et al., 2004), in which *Dscam* alone can generate up to 38,016 different isoforms through alternative splicing (Schmucker et al., 2000; Wojtowicz et al., 2004). Insects exhibit remarkable adaptability to their environment, and alternative splicing plays a crucial role in regulating various aspects of their adaptability. Regarding temperature adaptation, *D*. *melanogaster timeless* produces two isoforms under low temperature conditions, which regulate the circadian rhythm of *D. melanogaster* in that environment (Martin Anduaga et al., 2019). In the context of chemical sensing, there are at least eight different isoforms of acj6 in *Drosophila*, which play distinct roles in modulating odorant receptor genes (Bai and Carlson, 2010). In terms of immune response, the *Dscam* in *Anopheles gambiae* generates different isoforms through alternative splicing that can specifically bind to different pathogens, aiding in infection defense (Smith et al., 2011). Additionally, in the realm of pesticide resistance, the sodium ion channel gene in *Nilaparvata lugens* exhibits multiple isoforms, with significant variations in their sensitivity to neonicotinoid insecticides (Sun et al., 2020).

Sexual dimorphism arises from the long-term adaptation of insects to their environment, with alternative splicing playing a pivotal role in this adaptation. Within a single species, males and females share the majority of their genome, yet they often encounter divergent selective pressures (Andersson, 1994). These pressures can lead to significant sexual conflicts when they affect traits that have a shared genetic basis (Parker and Partridge, 1998; Bonduriansky and Chenoweth, 2009). Sex-biased alternative splicing serves as a mechanism to alleviate these conflicts, allowing for the retention of beneficial exons while splicing out harmful ones (Rogers et al., 2021). In the genomes of insects, there is a notable presence of sex-biased alternative splicing (Telonis-Scott et al., 2009; Hartmann et al., 2011; Brown et al., 2014). Isoform switching, generated by sex-biased alternative splicing events (Xing et al., 2020), plays an essential role in sex determination (Verhulst et al., 2010; Verhulst et al., 2013), development (Chang et al., 2011), and behavioral patterns (Sato et al., 2019). Therefore, the systematic understanding of sex-biased alternative splicing can hold promising prospects for pest control techniques. For instance, by manipulating genes involved in insect sex determination pathways through alternative splicing, researchers have developed female-specific release of insects carrying Dominant Lethals (fsRIDL) and precision guided Sterile Insect Technology (pgSIT) for pests control (Fu et al., 2007; Siddall et al., 2022). Additionally, targeting sex-specific alternative splicing of *Actin-4* has been used to weaken the flight ability of female mosquitoes for pest management (Labbé et al., 2012).

The accuracy of alternative splicing analysis is contingent upon the capability to accurately identify transcript splice sites and their specific combinations. Eukaryotic transcriptome analysis primarily relies on next-generation RNA sequencing (RNA-Seq) (Mutz et al., 2013), which involves extensive computational assembly of short reads. While RNA-Seq is adept at identifying splice sites, deducing the precise combinations of splice-site usage presents a significant challenge (Steijger et al., 2013; Wang et al., 2016). Technologies for full-length transcriptome sequencing, such as isoform sequencing (Iso-Seq), produce longer reads that eliminate the need for assembly, thus providing direct evidence of transcript structures. This advancement substantially enhances the accuracy of predicting splice site combinations in transcripts (Au et al., 2013; Tilgner et al., 2014; Treutlein et al., 2014; Gordon et al., 2015), outperforming RNA-Seq in the analysis of alternative splicing (Wang et al., 2016). Iso-Seq technology can overcome the presence of repeated sequences in insect genomes (Sproul et al., 2023), particularly addressing the issue of low accuracy in short-read assembly caused by tandem repeats (Tørresen et al., 2019). Presently, Iso-Seq has been employed in the analysis of alternative splicing events across various insects (Jia et al., 2018; Hong et al., 2020; Ouyang et al., 2021; Xing et al., 2022; Zheng et al., 2023).

The Oriental fruit fly (*Bactrocera dorsalis*) represents a major pest affecting fruit trees globally, inflicting damage on over 150 different types of fruit crops (Christenson and Foote, 1960). Male lures and protein baits are considered environmentally friendly control methods for managing *B. dorsalis*, taking advantage of the sexual dimorphism in its chemical sensing behavior (Tan and Nishida, 2012; Pagadala Damodaram et al., 2014; Zhang et al., 2023). Currently, in *B*. *dorsalis*, odorant receptor genes are the main molecular targets of its behavioral regulators (Liu et al., 2018); in addition, studies have found that other types of genes expressed in insect chemosensory organs are also involved in the behavioral regulation of insects. For instance, *takeout* influences insect feeding behavior, biological rhythms, male courtship behavior and chemoreceptive behavior (Sarov-Blat et al., 2000; Dauwalder et al., 2002; Bohbot and Vogt, 2005; Meunier et al., 2007); *P450* can serve as an odor-degrading enzyme and participate in the host plant recognition in *Locusta migratoria* (Wu et al., 2022); *CncC* can regulate the expression of odor-degrading enzymes in *L*. *migratoria* antennae (Wu et al., 2023). The antennae serve as the primary chemical sensory organs in insects, playing a key role in tactile sensing, olfaction, and taste (Lv et al., 2020). Thus, accurately and effectively analyzing alternative splicing events in the antennae is crucial for understanding the behavioral differences between males and females and for developing novel behavioral manipulation techniques for *B. dorsalis.* However, obtaining full-length transcriptome data from antennal tissues remains challenging due to the high RNA library requirements (>300 ng) for full-length transcriptome sequencing (Pacific Biosciences, 2023). Currently, most studies aiming to acquire full-length transcriptome data rely on pooling multiple samples or using tissues with larger volumes to gather sufficient RNA (Shields et al., 2021; Li et al., 2022). Despite these efforts, antennae, which have relatively lower RNA content, still lack comprehensive full-length transcriptome data specifically needed for research on behavioral molecular targets in *B. dorsalis*.

This study used the antennal full-length transcriptome and whole genome of *B. dorsalis* to systematically analyze sex-biased alternative splicing within its antennae. Initially, isoforms and gene loci in the *B. dorsalis* antennal full-length transcriptome were identified and quantified. Gene loci containing more than one isoform were then selected for further analysis. Subsequently, sex-biased alternative splicing events within these gene loci were annotated. Among the two most prevalent alternative splicing patterns, genes undergoing isoform switching, which exhibit differential protein-coding regions between female-biased and male-biased isoforms, were identified. These genes were categorized based on the length of the sex-biased isoforms. Additionally, within each category, isoform-switching genes that have the strongest sex preference and also have binding sites for transcription factors related to sexual differentiation in their upstream regulatory regions were further screened. Finally, the expression patterns of the screened genes and their conservation across closely related species were analyzed. These findings can provide a research foundation for understanding of functional differences between sexes in *B. dorsalis* genes regulated by alternative splicing and offer new insights for utilizing olfactory-guided behavioral control techniques in managing *B. dorsalis*.

## 2 Materials and methods

### 2.1 Insect rearing and sample collection

The population of *B. dorsalis* for this experiment was reared at the Shenzhen Institute of Genomics, Chinese Academy of Agricultural Sciences. The rearing conditions were 26 ± 1°C, 60%–70% relative humidity and a 14 L: 10 D (light: dark) cycle (darkness started at 20:00). The larvae were fed an artificial diet (Ren et al., 2021; Ren et al., 2023). Mature larvae were gathered and placed into small plastic jars filled with moist sand to allow for pupation. The pupae were then collected and transferred to Petri dishes until the adults emerged. Upon emergence, adults were separated by sex; the adult diet consisted of a 1:1 mixture of yeast and sugar in dry powder form, and a separate water container was provided. The female and male adults were reared until sexual maturity (12 days old) and then 500 individuals of each sex were collected for subsequent antennal tissue collection. The antennae were collected separately from both female and male *B. dorsalis*. In total, 500 pairs of female and male *B. dorsalis* antennae were collected, immediately frozen in liquid nitrogen, and stored at −80°C for ISO-Seq analysis (Supplementary Figure S1A).

### 2.2 Identification of full-length transcripts in *B.dorsalis* antennal full-length transcriptome and comparison with *B.dorsalis* antennal RNA-Seq transcripts

#### 2.2.1 Identification of non-redundant full-length transcripts in *B.dorsalis* antennal full-length transcriptome

The Iso-Seq library was prepared according to the Isoform Sequencing protocol (Iso-Seq) using the Clontech SMARTer PCR cDNA Synthesis Kit and the BluePippin Size Selection System protocol as described by Pacific Biosciences (PN 100-092-800-03). Sequence data were processed using the SMRTlink 6.0 software (https://www.pacb.com/support/software-downloads/). Circular consensus sequence (CCS) was generated from subread BAM files by CCS software, and the minimum quality was set to 0.9 (-min-rq 0.9). CCS. bam files were output, which were then classified into full length and non-full length reads using Lima, removing polyA using Isoseq3 refine. The Isoseq3 cluster program was used to obtain high-quality full-length consensus sequences. The Gmap version 2021-08-25 (Wu and Watanabe, 2005) was used to align the high-quality full-length consensus sequences to previously available *B. dorsalis* reference genome (National Bioinformatics Center GSA database, PRJCA020830) (parameters: -f samse -n 0 -B 5). The ToFu software in cDNA-Cupcake v29.0.0 (https://github.com/Magdoll/cDNA_Cupcake) was used to collapse redundant transcripts and merge female and male antennal full-Length transcriptome, obtaining non-redundant full-length transcripts of *B. dorsalis* antennae (Supplementary Figure S1B).

#### 2.2.2 Quality control and classification of non-redundant full-length transcripts in *B.dorsalis* antennal full-length transcriptome

Using Sqanti3 v3-5.1 (Tardaguila et al., 2018), combined with the previously available *B. dorsalis* reference genome and high-quality RNA-Seq data of *B. dorsalis* antennae, the *B. dorsalis* antennal non-redundant full-length transcripts were subjected to quality control, classification, and removal of potential artifacts (parameters: all_canonical: canonical, RTS_stage: FALSE, min_cov: 3), obtaining final full-length non-redundant transcript sequences and annotation files supported by *B. dorsalis* genome and *B. dorsalis* antennal RNA-Seq reads.

#### 2.2.3 Assembly of RNA-Seq reads of *B.dorsalis* antennae

Using Hisat2 v2.2.1 (Kim et al., 2019) default parameters, the high-quality RNA-Seq reads of *B. dorsalis* antennae were aligned to *B. dorsalis* reference genome, and the transcripts were assembled using Stringtie v2.2.1 (Pertea et al., 2015) (parameters: -rf -e) and Taco v0.6.2 (Niknafs et al., 2017) default parameters.

#### 2.2.4 Comparison of the length, GC content and splicing sites of non-redundant full-length transcripts and RNA-Seq transcripts in the antennae of *B.dorsalis*

According to the annotation files of full-length transcriptome and RNA-Seq transcriptome of *B. dorsalis* antennae, the gene loci detected by both transcriptome were screened using Gffread (Pertea and Pertea, 2020). The length and GC content of full-length transcripts and RNA-Seq transcripts in these gene loci were counted separately using Seqkit (Shen et al., 2016). The splicing sites of full-length transcripts and RNA-Seq transcripts in these gene loci were compared using Gffcompare (Pertea and Pertea, 2020).

### 2.3 Identification, functional enrichment analysis of multi-isoform genes in *B.dorsalis* antennal full-length transcriptome

#### 2.3.1 Functional annotation of genes identified in *B.dorsalis* antennal full-length transcriptome

Blastp v2.12.0 was used to compare all genes identified in *B. dorsalis* antennal full-length transcriptome with the NR library and the best alignment results of each gene were extracted. Interproscan v1.8.0 (Quevillon et al., 2005) (parameters: -dp -goterms -pa) was used to perform GO annotation on these genes; the KEGG website (https://www.genome.jp/kaas-bin/kaas_main) was used to perform KEGG annotation on these genes.

#### 2.3.2 Screening and functional enrichment analysis of mult-isoform genes in *B.dorsalis* antennal full-length transcriptome

Mult-isoform gene IDs were obtained based on the annotation file of the full-length transcriptome of the *B. dorsalis* antennae. Using the R package ClusterProfiler (Wu et al., 2021), all genes identified in the full-length transcriptome of *B. dorsalis* antennae were used as background to perform GO and KEGG functional enrichment analysis on multi-isoform genes. In the KEGG enrichment analysis, pathways with p. adjust <0.05 and qvalue <0.2 were defined as significantly enriched; in the GO enrichment analysis, GO terms with p. adjust <0.001 and qvalue <0.2 were defined as significantly enriched.

### 2.4 Identification of sex-biased alternative splicing events and isoform-switching genes in *B.dorsalis* antennal full-length transcriptome

#### 2.4.1 Quantification of non-redundant full-length transcripts in *B.dorsalis* antennal full-length transcriptome

Using the annotation files of *B. dorsalis* antennal full-length transcriptome and the high-quality RNA-Seq data of *B. dorsalis* antennae already available in the laboratory, we used Salmon v1.10.0 (Patro et al., 2017) quasi-mapping-based mode to quantify transcripts in *B. dorsalis* antennal full-length transcriptome.

#### 2.4.2 Identification of sex-biased alternative splicing and isoform-switching genes in *B.dorsalis* antennal full-length transcriptome

The R package IsoformSwitchAnalyzeR (Vitting-Seerup and Sandelin, 2019) was used to perform isoform switch analysis of multi-isoform genes identified in the full-length transcriptome of *B. dorsalis* antennae. The input to this package is a quantitative file from salmon, a full-length transcripts nucleotide sequence file, a full-length transcriptome annotation file, and a grouping file containing sample IDs and corresponding sexes. IsoformSwitchAnalyzeR evaluates isoform usage through isoform fraction (IF), which is the ratio of the expression level of a specific isoform to the expression level of its parent gene (isoform_expression/gene_expression). Differential isoform usage (DIU) is quantified as the difference in isoform fraction (dIF), calculated as IF_Female - IF_Male, to measure effect size. The IsoformSwitchTestDEXSeq () function was used to identify DIUs between sexes based on the dIF threshold. Isoforms that meet the criteria of |dIF| > 0.1, FDR <0.05, and TPM mean values greater than one in both females and males were defined as significantly sex-biased isoforms. If a gene contains at least two sex-biased isoforms with opposite effect sizes, it is considered to have undergone sex-biased isoform switching. The analyzeORF() function was used to obtain the longest open reading frame (ORF) of sex-biased isoforms, and the extractSequence () function was used to extract the nucleotide and amino acid sequences of the longest ORF of sex-biased isoforms. Based on the ORF nucleotide and amino acid sequences of sex-biased isoforms, CPC2 (http://cpc2.gao-lab.org/) was used to predict the coding potential of sex-biased isoforms, and the Pfam and SignalP databases in Interpro (https://www.ebi.ac.uk/interpro/search/sequence/) were used to predict the protein domains and signal peptides of sex-biased isoforms. IUPred3 (https://iupred.elte.hu/) was used to predict the intrinsically disordered regions (IDRs) of sex-biased splicing isoforms. The analyzeCPC2(), analyzeSignalP (), analyzePFAM(), and analyzeIUPred2A () functions were used to integrate the prediction results. If all sex-biased isoforms in an isoform switching gene have TPM mean values greater than 20 in at least one sex and there are differences in the protein coding regions between male-biased and female-biased isoforms, they were considered key isoform switching genes for subsequent analysis. The R package clusterProfiler was used to perform GO enrichment analysis on key isoform switching genes, and GO terms with p. adjust <0.05 and qvalue <0.2 were defined as significantly enriched.

### 2.5 The expression regulation prediction, expression pattern analysis and closely related species comparison of *BdorABCA13* and *BdorCAT2*

#### 2.5.1 Prediction of upstream transcription factor binding sites of *BdorABCA13*, *BdorCAT2* and *BdorTSN3*

For the genes *BdorABCA13*, *BdorCAT2*, and *BdorTSN3* identified within the *Bactrocera dorsalis* antennal full-length transcriptome, the transcription start site corresponding to the longest isoform was selected. The sequence from 5000bp upstream of the transcription start site to the start codon of the same isoform’s ORF was selected from the *B. dorsalis* reference genome sequence file as the candidate upstream regulatory region. The core transcription factor binding sites for *Drosophila melanogaster*, comprising 160 entries, were downloaded from the Jaspar database (https://jaspar2022.genereg.net/). PWMscan (Ambrosini et al., 2018) was used to align the *D. melanogaster* transcription factor binding sites to the candidate upstream regulatory region of each gene (*p*-value Cut-off = 1e-5). The amino acid sequence of the *D. melanogaster* transcription factors corresponding to the aligned transcription factor binding sites were found in the Uniprot database (https://www.uniprot.org/). Orthofinder v2.5.4 (Emms and Kelly, 2019) default parameters and Reciprocal Best Hit BLAST V.2 process (https://www.protocols.io/view/reciprocal-best-hit-blast-x54v9rezv3eq/) were used to find the homologous genes of the above-mentioned *D. melanogaster* transcription factors in the *B. dorsalis* reference genome. The genes with TPM >20 in female or male antennae were filtered as candidate transcription factor genes.

#### 2.5.2 Differential expression gene analysis of upstream transcription factor genes of *BdorABCA13*, *BdorCAT2* and *BdorTSN3* between sexes

According to the alignment results of *B. dorsalis* antennal RNA-Seq reads to *B. dorsalis* reference genome, combined with the annotation file of the *B. dorsalis* reference genome, FeatureCounts v2.0.1 (Liao et al., 2014) (parameters: -p -t CDS -g gene_id) was used to quantify the annotated genes in *B. dorsalis* reference genome, and only genes with TPM >0 were retained. The R package DESeq2 (Love et al., 2014) was used to perform differential expression gene analysis between female and male antennae, and genes that met the condition |log2FoldChange| > 0.58, Benjamini–Hochberg (BH) adjusted *p* < 0.05 were considered significantly differentially expressed genes between female and male antennae. The expression changes of the upstream transcription factor genes of *BdorABCA13*, *BdorCAT2* and *BdorTSN3* between female and male antennae of *B. dorsalis* were extracted from the differential expression gene analysis results.

#### 2.5.3 Analysis of the expression pattern of *BdorABCA13* and *BdorCAT2* in peripheral nervous tissues of *B.dorsalis*

The laboratory has previously obtained the RNA-Seq data of the peripheral nervous tissues of *B. dorsalis*, including the antennae, mouthparts, frontlegs, midlegs, hindlegs and genitalia of the female and male adult flies. Using Hisat2 v2.2.1 default parameters, the RNA-Seq reads of the peripheral nervous tissues of *B. dorsalis* were aligned to *B. dorsalis* reference genome, and the genes annotated in *B. dorsalis* reference genome were quantified using FeatureCounts v2.0.1 (parameters: -p -t CDS -g gene_id), and the raw counts and TPM values were calculated. The expression of *BdorABCA13* and *BdorCAT2* was extracted from the TPM expression matrix of the peripheral nervous tissues of *B. dorsalis*. The R package DESeq2 was used to perform differential expression gene analysis between sexes in the adult antenna, mouthparts, frontleg, midleg, hindleg and genitalia, and genes that met the condition |log2FoldChange| > 0.58, Benjamini–Hochberg (BH) adjusted *p* < 0.05 were considered significantly differentially expressed genes between female and male. The expression changes of *BdorABCA13*, *BdorCAT2* and *BdorTSN3* between female and male of *B. dorsalis* peripheral nervous tissues were extracted from the differential expression gene analysis results.

#### 2.5.4 Phylogenetic tree construction of ABCA13 and CAT2

The protein sequences of ABCA13 and CAT2 from *Anastrepha ludens*, *A. obliqua*, *B. cucurbitae*, *B. latifrons*, *B. neohumeralis*, *B. oleae*, *B. tryoni*, *Ceratitis capitata*, *Rhagoletis pomonella*, *R. zephyria* and *D. melanogaster* were collected from the NCBI database (https://www.ncbi.nlm.nih.gov/). These sequences were aligned using MAFFT v7.490 (Katoh et al., 2002) with the default parameters for multiple sequence alignment. Following the alignment, the maximum likelihood phylogenetic trees for ABCA13 and CAT2 were constructed separately using IQ-TREE v2.2.0.3 (Nguyen et al., 2015) with the parameters: -bb 1000 -nt 20 -m MFP --bnni.

#### 2.5.5 Reverse Transcription PCR (RT-PCR) validation of *BdorABCA13* and *BdorCAT2* isoforms

60 male adults and 60 female adults of *B. dorsalis* were selected, their antennae were collected between 9 a.m. and 11 a.m. (mixed samples of males and females), and the collected antennae were quickly frozen in liquid nitrogen. Total RNA was extracted from collected antennae using TriZol (Invitrogen, Carlsbad, CA, United States). Using approximately 1 µg of total RNA as a template, template cDNA of RT-PCR was synthesized using HiScript^®^ III first Strand cDNA Synthesis (+gDNAwiper) Kit (Vazyme, Nanjing, China). Adjust the concentration of template cDNA to about 500ng/ul and store at −20°C. According to transcriptome analysis results, both *BdorABCA13* and *BdorCAT2* have two isoforms, and there are distinct differences in the 5′regions between different isoforms of the same gene. However, the Open Reading Frame (ORF) and 3′ Untranslated Region (3′ UTR) sequences and structures exhibit high similarity (Figures 4B, C). To design specific primers, in each isoform, we set the forward primer in the 5′UTR and the reverse primer in the ORF, and verified the existence of alternative splicing by detecting the 5′region of the isoform with obvious differences (Figure 5A; Supplementary Table S4). The specific primers for 5’ region of each isoform were used for amplification under the following conditions: 98°C for 3 min; followed by 35 cycles of denaturation at 98°C for 10 s, annealing at 60°C for 15 s, and extension at 72°C for 1 min; with a final extension step at 72°C for 10 min; the samples were then held at 12°C. The PCR products of each isoform were then cloned into blunt-end vectors, and 24 single-clonal colonies were screened from each product to identify whether the PCR cloned sequence is consistent with the sequence obtained from transcriptome analysis.

#### 2.5.6 Quantitative Real-Time PCR (qRT-PCR) validation of *BdorABCA13* and *BdorCAT2* isoform switch in *B.dorsalis* antennae

60 male adults and 60 female adults of *B. dorsalis* were selected, their antennae were collected between 9 a.m. and 11 a.m. (separated samples of males and females). and the collected antennae were quickly frozen in liquid nitrogen. Total RNA was extracted from collected antennae using TriZol (Invitrogen, Carlsbad, CA, United States). cDNA was synthesized using about 1 µg of total RNA as a template and HiScript^®^ III first Strand cDNA Synthesis (+gDNAwiper) Kit (Vazyme, Nanjing, China) as a qRT-PCR template, and stored at −20°C (Supplementary Figure S5A). Based on the transcript sequences obtained from transcriptome analysis, NCBI Primer-BLAST (https://www.ncbi.nlm.nih.gov/tools/primer-blast/index.cgi?LINK_LOC=BlastHome) was used to design specific primers, and *BdorActin2* were used as endogenous control (Shen et al., 2010) (Supplementary Table S5). The PCR volume was 10 μL, including 5 μL 12× Taq Pro Universal SYBR qPCR Master Mix (Vazyme, Nanjing, China), 0.5 μL of each primer (10 μM), 1 μL cDNA, and 4 μL RNase-free water. PCR was performed on a CFX96 real-time PCR detection system (Bio-Rad, Hercules, CA). Before formal experiments, dissociation curve analysis was performed to ensure the specificity of amplification (Supplementary Figure S5B; Supplementary Table S6). Relative gene expression levels for each sample were calculated using the 2^−ΔΔCT^ method (Livak and Schmittgen, 2001). A total of three biological replicates were performed. Statistical analysis of qPCR results was performed using Graph Pad Prism (Version 8.0.1). All replicate data results are expressed as mean ± standard error. The original data were first tested for normality using the Shapiro-Wilk test, and non-normally distributed data were transformed using log (x+1). Unpaired t-test was used to compare the expression levels of *BdorABCA13* and *BdorCAT2* isoforms in the male and female antennae of *B. dorsalis*. The difference is significant when <0.05.

## 3 Results

### 3.1 Identification of full-length transcripts in *B.dorsalis* antennal full-length transcriptomes and comparison with RNA-Seq transcripts

**FIGURE 1:**
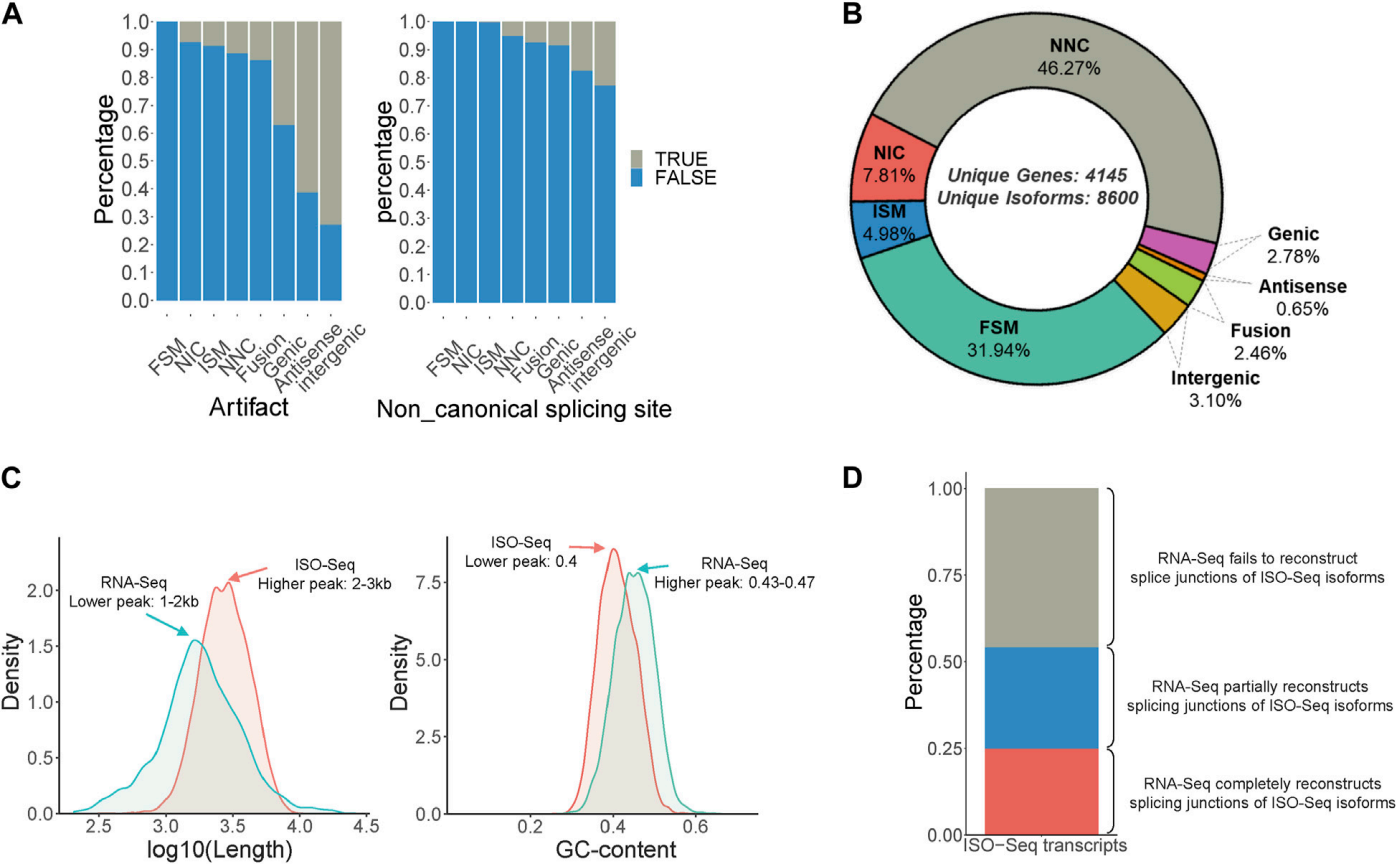
Identification of full-Length transcripts in *B. dorsalis* antennal full-length transcriptome. **(A)** Quality control of non-redundant full-length transcripts in *B. dorsalis* antennal full-length transcriptome. Quality control metrics include artifact proportion and non-canonical splicing junction proportion. **(B)** Classification of non-redundant full-length transcripts and the proportion of each category. FSM: Matches all SJ perfectly. ISM: Matches the reference SJs partially. NIC: Novel isoform with a new combination of known splice sites. NNC: Novel isoform with at least a new splicing site. Antisense: Novel isoform is anti-sense to an annotated gene. Genic: Overlaps introns and exons. Intergenic: Novel isoform is in the intergenic region. **(C)** The distribution of length and GC content of ISO-Seq transcripts and RNA-Seq transcripts among the gene loci detected by both ISO-Seq transcriptome and RNA-Seq transcriptome of *B. dorsalis* antennae. **(D)** The proportion of ISO-Seq transcripts that can be reconstructed by RNA-Seq transcripts in gene loci detected by both RNA-Seq transcripts and ISO-Seq transcripts. RNA-Seq completely reconstructs splicing junctions of ISO-Seq isoforms means that the number of exons and splicing junctions of transcripts reconstructed by RNA-Seq reads are completely consistent with ISO-Seq transcripts; RNA-Seq partially reconstructs splicing junctions of ISO-Seq isoforms means that the number of exons of transcripts reconstructed by RNA-Seq reads is less than that of ISO-Seq transcripts, but the splicing junctions are consistent with ISO-Seq transcripts; RNA-Seq fails to reconstruct splice junctions of ISO-Seq isoforms means that the splicing junctions of transcripts reconstructed by RNA-Seq reads are inconsistent with ISO-Seq transcripts.

Isoform sequencing was performed using RNA obtained from the male and female antennae of *B. dorsalis*, and the original subreads obtained were 18,374,257 and 12,437,865, respectively, each with a length exceeding 2500 bp. These reads were then processed using Isoseq3 workflow, involving steps such as adapter removal, classification, and clustering, resulting in 10,218 and 6,893 full-length (FL) reads, respectively (Supplementary Table S1). The FL reads were then aligned to the *B. dorsalis* reference genome using Gmap. Redundancy was further removed using Cupcake ToFU, and artifacts were eliminated using SQANTI3. In total, 8,600 non-redundant full-length transcripts were obtained, covering 4,145 gene loci (Supplementary Table S7). These transcripts can be categorized into eight types, ranked from highest to lowest proportion: Novel isoform with at least one new splicing site (NNC), Matches all splicing sites perfectly (FSM), Novel isoform with a new combination of known splice sites (NIC), Matches the reference splicing sites partially (ISM), Novel isoform in the intergenic region (Intergenic), Fusion, Overlaps introns and exons (Genic), and Novel isoform antisense to an annotated gene (Antisense) (Figures 1A, B). Additionally, a comparison between the ISO-Seq transcriptome and the RNA-Seq transcriptome for shared gene loci revealed that the ISO-Seq transcriptome had a peak transcript length around 2–3 kb, which is longer than the 1–2 kb observed in the RNA-Seq transcriptome (Figure 1C). Regarding GC content, ISO-Seq transcriptome had a peak value of 0.4, slightly lower than the 0.43-0.47 observed in RNA-Seq transcriptome (Figure 1C). In terms of splice junction structures, ISO-Seq transcriptome provided more accurate combinations of splice sites, with only 24.8% of ISO-Seq transcriptome junctions fully reconstructed by RNA-Seq reads, an additional 29.3% were partially reconstructed, and over 40% of the combinations remained unreconstructed (Figure 1D). In summary, utilizing antennal ISO-Seq transcriptome in conjunction with the genome enables effective discovery of novel alternative splicing events and significantly enhances the quality of identified transcripts.

### 3.2 Identification and functional annotation of multi-isoform genes, and analysis of alternative splicing in odorant receptor genes within the *B.dorsalis* antennal full-length transcriptome

**FIGURE 2:**
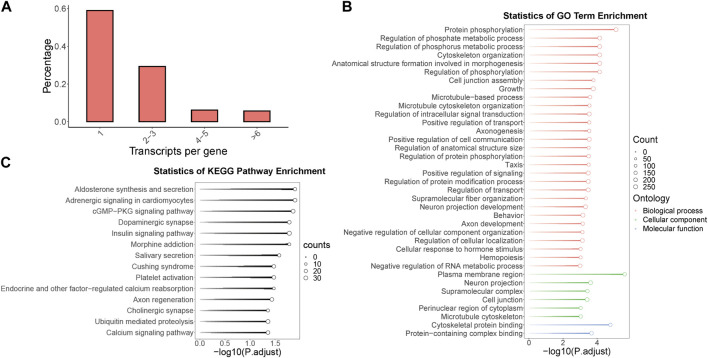
The identification, functional annotation of multi-isoform genes in *B. dorsalis* antennal full-length transcriptome. **(A)** The distribution of isoform numbers in gene loci identified by the full-length transcriptome of *B. dorsalis* antennae. **(B)** KEGG enrichment analysis of multi-isoform genes. **(C)** GO enrichment analysis of multi-isoform genes.

Within the context of the full-length transcriptome of *B. dorsalis* antennae, we screened for genes with multiple isoforms and identified a total of 1,704 genes, which account for 41.1% of all gene loci. Remarkably, some single genes were found to contain up to 45 isoforms. Among these genes, the highest proportion consists of single genes containing two to three isoforms, representing 29.3% of all gene loci (Figure 2A). To further understand the potential role of these multi-isoform genes in the physiological processes of *B. dorsalis*, we performed functional annotation on the genes identified from the antennal full-length transcriptome through GO enrichment analysis (with a significance level of *p* < 0.001) and KEGG enrichment analysis (with a significance level of *p* < 0.05) specifically for the multi-isoform genes (Supplementary Figure S2A). The GO enrichment results revealed that multi-isoform genes significantly enriched in 37 GO terms. Among these, 29 were related to biological processes, with protein phosphorylation being the most prominent. Additionally, six terms were associated with cellular components, with the plasma membrane region being the most enriched. Two terms were related to molecular functions, with the most significant enrichment in cytoskeletal protein binding (see Figure 2B). Furthermore, the KEGG enrichment analysis demonstrated that multi-isoform genes significantly enriched in 14 KEGG pathways, including pathways related to neuronal functions, such as dopaminergic synapse, cholinergic synapse, and calcium signaling pathway (Figure 2C). Multi-isoform genes obtained from *B. dorsalis* antennal full-length transcriptome were quantified using *B. dorsalis* antennal RNA-Seq reads, and alternative splicing analysis were performed on these genes using exon-based method. In total, we annotated 1,602 genes that exhibit alternative splicing events, covering seven types of such events: alternative 3′ end (A3), alternative 5’ end (A5), alternative transcription start sites (ATSS), alternative transcription termination site (ATTS), exon skipping (ES), intron retention (IR), and mutually exclusive exons (MES) (Supplementary Table S8). Regarding odorant receptor (OR) genes, we identified two OR genes (*BdorOR63a.1* and *BdorOR10a* and the odorant co-receptor (ORCO) gene *BdorORCO* have multiple isoforms. *BdorOR63a.1* was identified with three isoforms, one of which underwent RI and A5 alternative splicing events without altering the protein coding regions (Supplementary Figure S3A). *BdorOR10a* was identified with three isoforms, and two of these isoforms exhibited RI and A5 alternative splicing events compared to the constitutive splicing transcript (Supplementary Figure S3B). *BdorORCO* was identified with nine isoforms, revealing A3, A5, RI, and SE alternative splicing events, resulting in three distinct protein coding regions (Supplementary Figure S3C).

### 3.3 Identification of sex-biased alternative splicing and isoform-switching genes in *B.dorsalis* antennal full-length transcriptome

**FIGURE 3:**
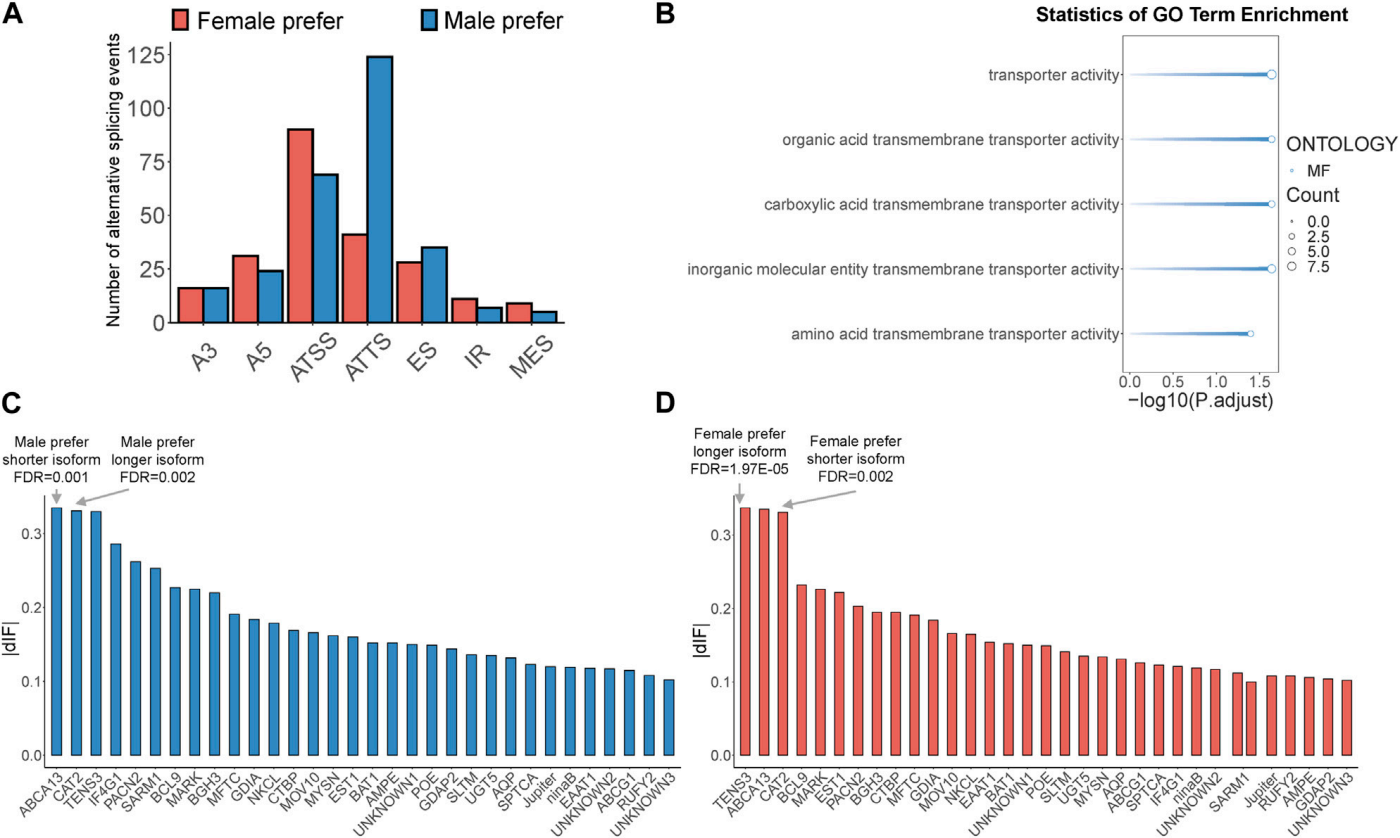
Identification of sex-biased alternative splicing and isoform-switching genes in *B. dorsalis* antennal full-length transcriptome. **(A)** The distribution of sex-biased splicing events in isoform-switching genes. A3: Alternative 3′ acceptor site. A5: Alternative 5′ donor site. ATSS: Alternative Transcription Start Site. ATTS: Alternative Transcription Termination Site. ES: Exon Skipping. IR: Intron Retention. MES: Multiple Exon Skipping. **(B)** GO enrichment analysis of key isoform-switching genes. **(C)** The distribution of dIF values of male-biased isoforms in key isoform-switching genes. **(D)** The distribution of dIF values of female-biased isoforms in key isoform-switching genes.

Sex-biased alternative splicing was screened based on the differences in isoform splicing sites and expression patterns between males and females. A total of 775 sex-biased alternative splicing events were annotated, of which 506 produced isoform switches, covering 161 gene loci. These isoform switches were attributed to seven types of alternative splicing: A3, A5, ATSS, ATTS, ES, IR, and MES (Supplementary Table S9). Notably, ATSS and ATTS were the most prevalent, accounting for 31% and 33% of all sex-biased alternative splicing events, respectively, with ATSS being more common in females and ATTS more common in males (Figure 3A). Among the 161 genes mentioned above, we further screened genes with high expression of sex-biased alternative splicing transcripts (TPM>20) and sex-biased ATSS or ATTS alternative splicing occurring in the protein coding region as key isoform-switching genes for subsequent analysis, and finally obtained 32 key genes (Supplementary Table S9). To determine the potential functions of key isoform-switching genes, we performed GO enrichment analysis on these genes. The results showed that the five significantly enriched GO terms were all related to transporter activity (Figure 3B). According to the length of sex-biased isoforms, we divided the above 32 genes into four groups: male prefer shorter isoform, male prefer longer isoform, female prefer shorter isoform, and female prefer longer isoform. We ranked the male-biased and female-biased isoforms among the 32 genes by descending dIF value and identified the genes with the strongest sex-biased isoform in each of the four categories. We found that the genes with the strongest sex bias in these categories all involved ATSS alternative splicing. The gene with the strongest male-biased isoform in the case of male prefer shorter isoform was *ATP-binding cassette sub-family A member 13*, *BdorABCA13*, and the gene with the strongest male-biased isoform in the case of male prefer longer isoform was *Cationic amino acid transporter 2*, *BdorCAT2* (Figure 3C); the gene with the strongest female-biased isoform in the case of female prefer shorter isoform was *Cationic amino acid transporter 2*, *BdorCAT2*, and the gene with the strongest female-biased isoform in the case of female prefer longer isoform was *Tension-3*, *BdorTSN3* (Figure 3D).

### 3.4 Prediction of expression regulation, analysis of expression patterns, and comparison with closely related species for *BdorABCA13* and *BdorCAT2*

**FIGURE 4:**
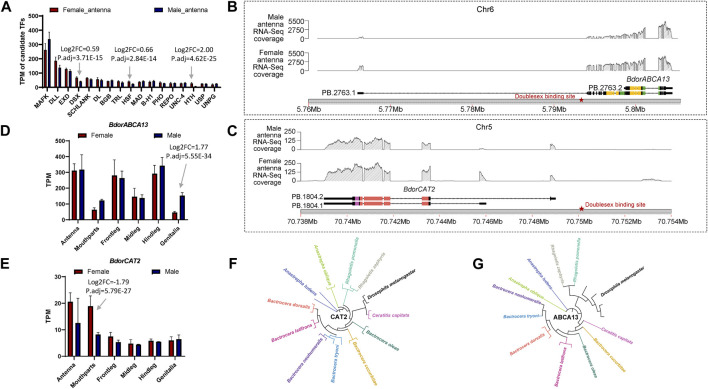
The expression regulation prediction, expression pattern analysis and closely related species comparison of *BdorABCA13* and *BdorCAT2*. **(A)** Statistics of the TPM values of the transcription factors that bind to the upstream regulatory regions of *BdorABCA13* and *BdorCAT2*. *DSX*, *HSF* and *HTH* have significant differences in expression between female and male. **(B)** Isoform structure, RNA-Seq coverage and location on *B. dorsalis* reference genome of the two isoforms of *BdorABCA13*. Orange represents the ABC2-membrane-3 domain. Green represents the ABC-tran-domain. Red stars indicate the predicted doublesex transcription factor binding sites. **(C)** Isoform structure, RNA-Seq coverage and location on *B. dorsalis* reference genome of the two isoforms of *BdorCAT2*. Purple represents the AA-permease-C domain. Red represents the AA-permease-2 domain. Red stars indicate the predicted doublesex transcription factor binding sites. **(D)** The expression pattern of *BdorABCA13* in *B. dorsalis* peripheral nervous tissues. **(E)** The expression pattern of *BdorCAT2* in *B. dorsalis* peripheral nervous tissues. **(F)** The phylogenetic tree of *D. melanogaster* CAT2 and CAT2 in 11 Tephritidae species. **(G)** The phylogenetic tree of *D. melanogaster* ABCA13 and ABCA13 in 11 Tephritidae species.

**FIGURE 5:**
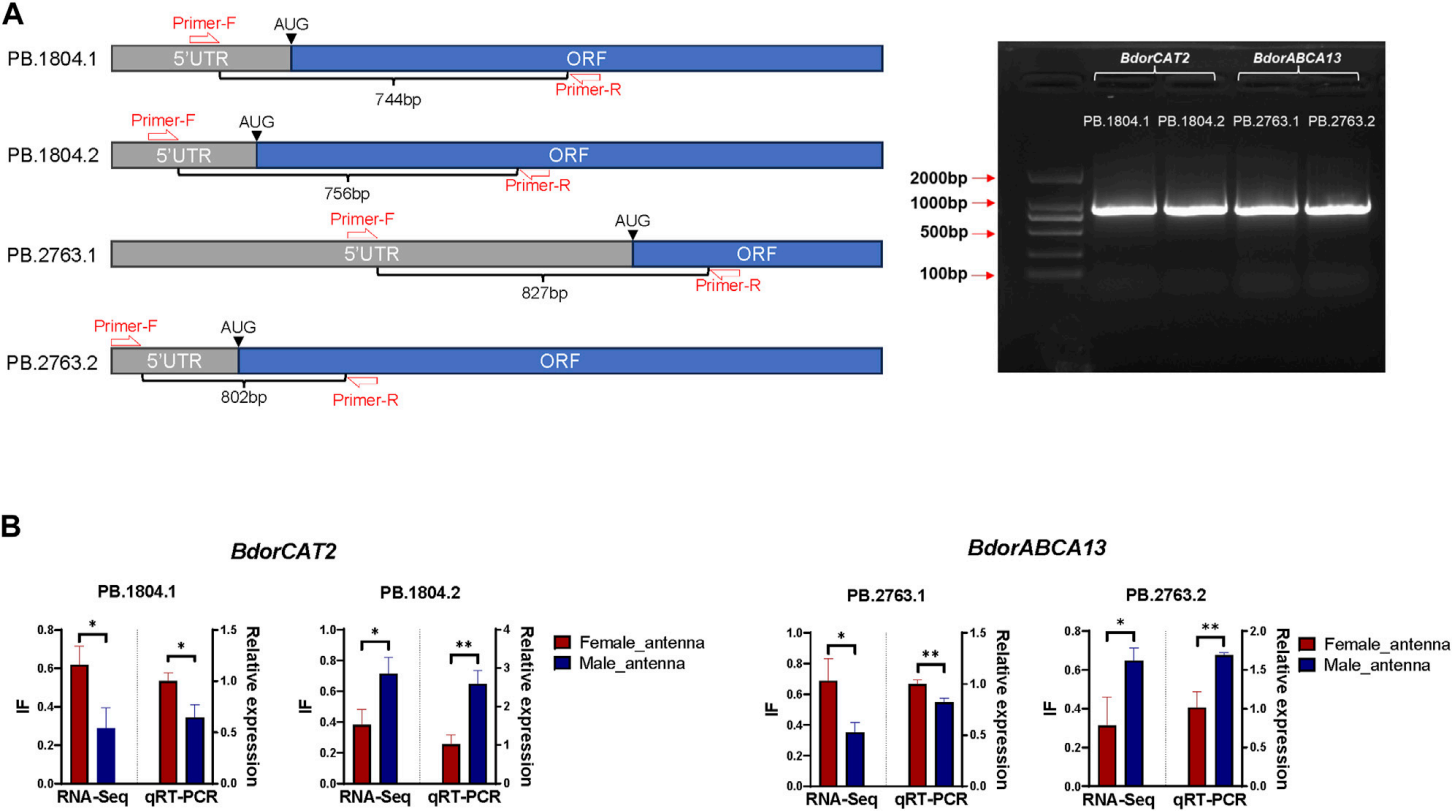
Validation of isoform switch of *BdorCAT2* and *BdorABCA13*. **(A)** Reverse Transcription PCR (RT-PCR) primer setting positions and gel map of *BdorCAT2* and *BdorABCA13* isoforms. **(B)** RNA-Seq quantification and Quantitative Real-Time PCR (qRT-PCR) verification of *BdorCAT2* and *BdorABCA13* isoforms in male and female antennae of *B. dorsalis*.

To further elucidate the relationship between the identified key isoform-switching genes and regulation related to sexual differentiation, we predicted transcription factors in the upstream regulatory regions of *BdorABCA13*, *BdorCAT2*, and *BdorTSN3*. The transcription start site corresponding to the longest isoform, as derived from the *B. dorsalis* antennal full-length transcriptome for each gene, was selected. The sequence from 5000 bp upstream of the transcription start site to the start codon of the same isoform’s ORF was extracted from the *B. dorsalis* reference genome sequence file as the candidate upstream regulatory region. Using the transcription factor binding sites information of *D. melanogaster* in the public database, the analysis was performed according to the workflow shown in the figure (Supplementary Figure S1C). A total of 17 transcription factor binding sites were identified (Supplementary Table S3), among which only doublesex is associated with sex-specific differentiation, exhibiting the highest expression level among the three sex-biased transcription factors (padj = 3.71E-15, logFC = 0.595) (Figure 4A; Supplementary Table S10). The doublesex binding site was found exclusively in the upstream regions of *BdorABCA13* and *BdorCAT2*, suggesting that the sex-biased alternative splicing of these two genes might be regulated by doublesex. The upstream doublesex binding site of *BdorABCA13* is located at 336bp-347bp upstream of the start codon of the longest isoform, and is located in the intron of the 5′UTR (Figure 4B). The upstream doublesex binding site of *BdorCAT2* is located at 248bp-259bp upstream of the transcription start site of the longest isoform (Figure 4C). To validate the alternative splicing and isoform switches of *BdorABCA13* and *BdorCAT2*, we confirmed the presence of their isoform structures using RT-PCR. Additionally, we quantified the expression of isoforms of these two genes in the female and male antennae of *B. dorsalis* using qRT-PCR. Given the high similarity between ORF regions and 3′ UTR regions of the isoforms of these two genes, we selectively amplified the 5′ end fragment with noticeable differences. The results revealed that all isoforms of *BdorABCA13* and *BdorCAT2* exist and exhibit significant differences between males and females, consistent with the findings from the transcriptome analysis (Figures 5A, B). *BdorABCA13* was expressed in all peripheral nervous tissues, and had relatively high expression level in the antennae, forelegs and hindlegs; in the genitalia, *BdorABCA13* had significantly higher expression in male than in female (log2FoldChange = 1.77, P. adj = 5.55E-34), and no significant sex difference in other peripheral nervous tissues (Figure 4D; Supplementary Table S10). *BdorCAT2* was expressed in all peripheral nervous tissues, and had the highest expression level in the antennae; in the mouthparts, *BdorCAT2* had significantly higher expression in female than in male (log2FoldChange = −1.79, P. adj = 5.79E-27), and no significant sex difference in other tissues (Figure 4E; Supplementary Table S10). Therefore, we speculate that *BdorABCA13* and *BdorCAT2* can play roles in multiple peripheral nervous tissues. To verify whether the alternative splicing of these two genes is conserved among the Tephritidae species, we collected the protein sequence data of the above two genes from 11 Diptera species (10 Tephritidae species and *D*. *melanogaster*) from the NCBI database (Supplementary Table S2), and constructed phylogenetic trees respectively. The results showed that except for *R. zephyria*, *CAT2* of other Tephritidae species and *D*. *melanogaster* had two isoforms, and the splicing type was ATSS (Figure 4F; Supplementary Figure S4A); for *ABCA13*, *D*. *melanogaster* had four isoforms; in Tephritidae species, only *B. dorsalis* and *B. correcta* had two isoforms of *ABCA13*, and the alternative splicing type was ATSS, while other Tephritidae species had only one isoform (Figure 4G; Supplementary Figure S4B).

## 4 Discussion

Alternative splicing contributes to the diversification of gene functions and is prevalent in insects displaying sexual dimorphism. Several studies have comprehensively annotated the sex-biased alternative splicing events in *Drosophila* species using microarrays and next-generation sequencing technologies (McIntyre et al., 2006; Chang et al., 2011; Singh and Agrawal, 2023), revealing that this phenomenon is evolutionarily conserved (Gibilisco et al., 2016). Chemoreception plays a crucial role in the environmental adaptation of *B. dorsalis*, with reported sexual dimorphism in chemosensory behavior. For example, methyl eugenol can only attract *B. dorsalis* males, and benzothiazole and 1-octen-3-ol can induce oviposition behavior of female *B. dorsalis* after mating, with potential molecular targets for behavioral regulation identified through these phenomena (Miyazaki et al., 2018; Xu et al., 2023). However, current research primarily focuses on differences in gene expression between sexes and has not extensively explored post-transcriptional regulatory processes such as alternative splicing. The antennae serve as the primary chemosensory organs in *B. dorsalis*. Investigating sex-biased alternative splicing in the antennae of *B. dorsalis* will enhance our understanding of the molecular mechanisms underlying chemosensory behavioral dimorphism and identify new molecular targets for behavioral regulation in *B. dorsalis*. This study successfully acquired the full-length antennal transcriptome of *B. dorsalis* through isoform sequencing technology and created a comprehensive, non-redundant full-length transcripts of *B. dorsalis* antennae by integrating next-generation RNA sequencing, isoform sequencing, and bioinformatics methods. The full-length transcriptome was utilized to thoroughly annotate sex-biased alternative splicing events and isoform-switching genes in *B. dorsalis* antennae, and to predict the upstream transcription factors of *BdorABCA13* and *BdorCAT2*, which experienced ATSS type sex-biased alternative splicing. This research provides high-quality ISO-Seq data of *B. dorsalis* antennae and establishes a foundation for the development of new behavioral manipulation technologies for *B. dorsalis*.

Previous studies have shown that tissue-specific full-length transcriptome can improve the quality of genome annotation. For example, in the full-length transcriptome of silkworm silk gland and human bone marrow, 77% and 60%–70% of transcript structures not identified in the original genome annotation, respectively (Deslattes Mays et al., 2019; Chen et al., 2020). In this study, 31.94% of the full-length transcripts from the *B. dorsalis* antennal full-length transcriptome perfectly matched all splicing sites with the reference genes in the *B. dorsalis* reference genome annotation. The remaining approximately 68% were not identified in the current genome annotation file, suggesting that these transcripts represent either new transcripts of known genes or entirely new genes. This indicates that compared to the existing genome annotation, utilizing the antennal full-length transcriptome allows for a more accurate acquisition of the transcript structure of genes expressed in the antennal tissue, which aids in inferring their potential functions. Among all the new transcripts identified by the *B. dorsalis* antennal full-length transcriptome, those classified as NNC type transcripts constituted the highest proportion. This suggests that, compared to the *B. dorsalis* reference genome annotation, the antennal full-length transcriptome revealed more new types of splice sites. This may be caused by two reasons: firstly, the *B. dorsalis* reference genome annotation used in this study did not provide the structure information of the gene UTR region, while the transcripts obtained by the antennal full-length transcriptome retained the UTR region, and the gene structure obtained was more complete; secondly, for a single gene locus, the antennal full-length transcriptome can obtain more comprehensive alternative splicing transcript structures. Having complete information on gene and transcript structures provides a solid foundation for researching gene functions.

Previous studies have indicated that transcripts derived from full-length transcriptome technologies like ISO-Seq offer superior advantages in terms of length and structural integrity compared to those obtained from RNA-Seq technology (Sharon et al., 2013). This study screened the gene loci detected by both antennal RNA-Seq transcriptome and antennal ISO-Seq transcriptome of *B. dorsalis*, and compared the RNA-Seq transcripts and ISO-Seq transcripts in these gene loci from three aspects: transcript length, GC content and splice junction structure. In terms of transcript length, the length of transcripts in ISO-Seq transcriptome was longer than that of RNA-Seq transcripts, which was consistent with previous studies in insects, such as ISO-Seq transcripts of *Agasicles hygrophila*, about 39% of which were longer than 2kb, while only 2.31% of RNA-Seq transcripts were longer than 2 kb (Jia et al., 2018); in terms of GC content, the GC content of ISO-Seq transcripts was slightly lower than that of RNA-Seq transcripts, and previous studies have shown that the GC content of gene UTR regions was lower than that of CDS regions (Jaksik and Rzeszowska-Wolny, 2012), and ISO-Seq transcripts could obtain more complete UTR region structures, which might lead to lower average GC content of ISO-Seq transcripts; in terms of splice junction structure, only about 24.8% of ISO-Seq transcripts had splice junction structures that were completely consistent with RNA-Seq transcripts, which might be caused by misassembled transcripts generated during the RNA-Seq transcript assembly process (Steijger et al., 2013). Correct splice structure is essential for gene function research, and ISO-Seq can provide more accurate structural information for gene function research than RNA-Seq.

Several previous studies have reported alternative splicing events of insect odorant receptor genes, which may imply that isoforms have different roles in olfactory-guided behavioral responses (Robertson et al., 2003; Cattaneo, 2018; Garczynski et al., 2019). This study found that *BdorOR63a.1* and *BdorOR10a*, two olfactory receptor genes, undergo alternative splicing. The relationship between alternative splicing of odorant receptor genes and their functional changes is still poorly understood, but some studies have found that changes in the C-terminal of amino acid sequence of odorant receptors affect ligand specificity (Hill et al., 2015); in addition, this study also found that the odorant co-receptor gene *BdorORCO* undergoes alternative splicing, and previous studies have found that *ORCO* in *D. melanogaster* also has two isoforms, but the protein coding regions of the two isoforms are the same (Matthews et al., 2015). The relationship between ORCO structure and function is still unknown, but its C-terminal structure is thought to be involved in receptor-receptor protein interactions (Miller and Tu, 2008). The impact of alternative splicing on the function of odorant receptor genes and odorant co-receptor genes warrants further experimental investigation.

Alternative transcription start sites (ATSS) are important forms of alternative splicing, which can further assist post-transcriptional regulation by affecting mRNA stability and protein translation efficiency, and are widely present in the genomes of many species (Rach et al., 2009; Yoon et al., 2019). ATSS can affect important life activities of insects. ATSS may be related to insect growth and development, such as the alcohol dehydrogenase (Adh) gene of *D. melanogaster* uses different promoters to produce two isoforms with different transcription start sites, one mainly expressed in the embryo and larval stages, and the other mainly expressed in the adult stage (Corbin and Maniatis, 1989); the *Hunchback* (*Hb*) of *D. melanogaster* also produces two isoforms with different transcription start sites, and they play roles in different stages of embryonic development (Margolis et al., 1995). This study identified 506 sex-biased alternative splicing events in the antennal tissue of *Bactrocera dorsalis*, with ATSS accounting for 31% of these events. This phenomenon of ATSS having a relatively high proportion in alternative splicing events has also been found in other species (Reyes and Huber, 2018; Chen et al., 2022). Currently, there are a lack of studies on the relationship between ATSS events and inter-sexual differentiation of gene functions in insects. However, we speculate that ATSS may play a role in the sex-specific development process of *B. dorsalis* antennal tissue, though its precise function requires further investigation.

In insects, *doublesex* coordinate regulatory network of genes (Arbeitman et al., 2016), and this network is thought to be related to sexual dimorphism in insects (Lee et al., 2002; Iijima et al., 2019; Han et al., 2022). In this study, we found binding sites of doublesex transcription factors in the upstream regulatory regions of both *BdorABCA13* and *BdorCAT2* genes. Therefore, we speculate that the alternative splicing of these two genes may be regulated by doublesex. ATP-binding cassette transporters are divided into full-transporters and half-transporters. Full-transporters have two nucleotide-binding domains and two transmembrane domains, and can function directly; half-transporters only contain one nucleotide-binding domain and one transmembrane domain, and can only function when forming homologous or heterologous dimers with another half-transporter (Hou et al., 2000; Jones and George, 2004; Hollenstein et al., 2007). This study found that the female-biased isoform of *BdorABCA13* is a full-transporter, while the male-biased isoform is a half-transporter (Figure 4C). In fruit flies, this gene is thought to be involved in lipid transport (Dermauw and Van Leeuwen, 2014). Studies have shown that there are differences in lipid metabolism between female and male insects. During adult development, females need to consume a large amount of lipids to generate oocytes, while males need to store a lot of lipids for flight energy (Gilbert and Chino, 1974). Therefore, we speculate that the sex-biased isoform switch of *BdorABCA13* is related to the differences in lipid metabolism between females and males. The full-transporter form in females can significantly improve the lipid transport efficiency, to meet the high lipid metabolism demand of female *B. dorsalis*. *BdorCAT2* gene contains two isoforms, both isoforms have no difference in domain structure, but the female-biased isoform has a shorter first intron than the male-biased isoform (Figure 4B). Long introns increase the transcription cost of genes (Castillo-Davis et al., 2002; Swinburne et al., 2008). Studies have found that CAT2 is related to the nutritional signal transduction in the reproductive process of female insects (Attardo et al., 2006). We speculate that the shorter first intron of the female-biased isoform of *BdorCAT2* is to enhance the transcription efficiency of the gene, so that it can play a faster role in the nutritional signal transduction.

This study used the full-length transcriptome and whole genome of *B. dorsalis* to systematically analyze sex-biased alternative splicing within its antennae. First, isoforms and gene loci were identified and quantified in the full-length transcriptome of the *B. dorsalis* antennae. Gene loci containing more than one isoform were then selected for further analysis. Next, sex-biased alternative splicing events within these gene loci were annotated. Among the two most prevalent alternative splicing patterns, isoform-switching genes that have differential protein-coding regions between female-biased and male-biased isoforms were identified, and they were categorized based on the length of the sex-biased isoforms. Additionally, *BdorABCA3* and *BdorCAT2* were screened from the above isoform-switching genes, both of which have doublesex transcription factor binding sites upstream of the genes. Finally, the expression patterns and conservation among closely related species of these two genes were analyzed. This study provided high-quality full-length transcriptome data of *B. dorsalis* antennae, which laid the foundation for the development of novel behavioral regulators of *B. dorsalis*.

## Data Availability

The data presented in this study can be found in the Genome Sequence Archive repository (https://ngdc.cncb.ac.cn/gsa/), accession number CRA015403.
