# Supplementary material for: Identification of antennal alternative splicing by combining genome and full-length transcriptome analysis in Bactrocera dorsalis
Source: Front Physiol. 2024 Jun 17;15:1384426. doi: 10.3389/fphys.2024.1384426 (PMC11215311; doi:10.3389/fphys.2024.1384426)
Supplement: Supplementary file 9 [file DataSheet1.docx]

Supplementary Figures

# Supplementary Figures

**
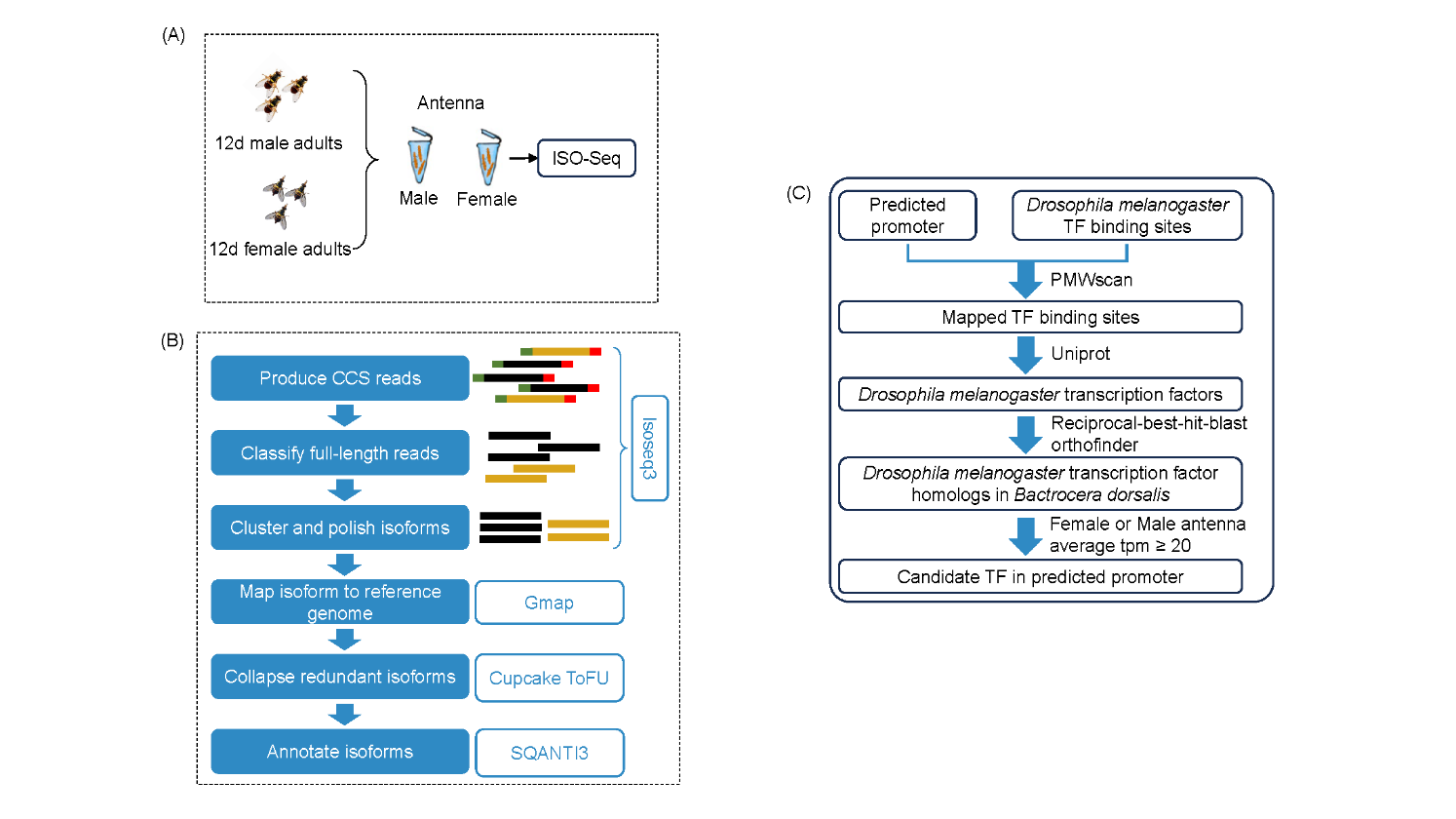
**

**Supplementary Figure 1.** The sample collection scheme and analysis procedures used in this study. (A) The sample collection scheme of *B.dorsalis* antennae. (B) Bioinformatics pipeline to reconstruct the *B.dorsalis* antennal full-length transcriptome from Iso-Seq dataset. (C) The prediction method of transcription factors in the upstream regulatory regions of *BdorABCA13* and *BdorCAT2*.


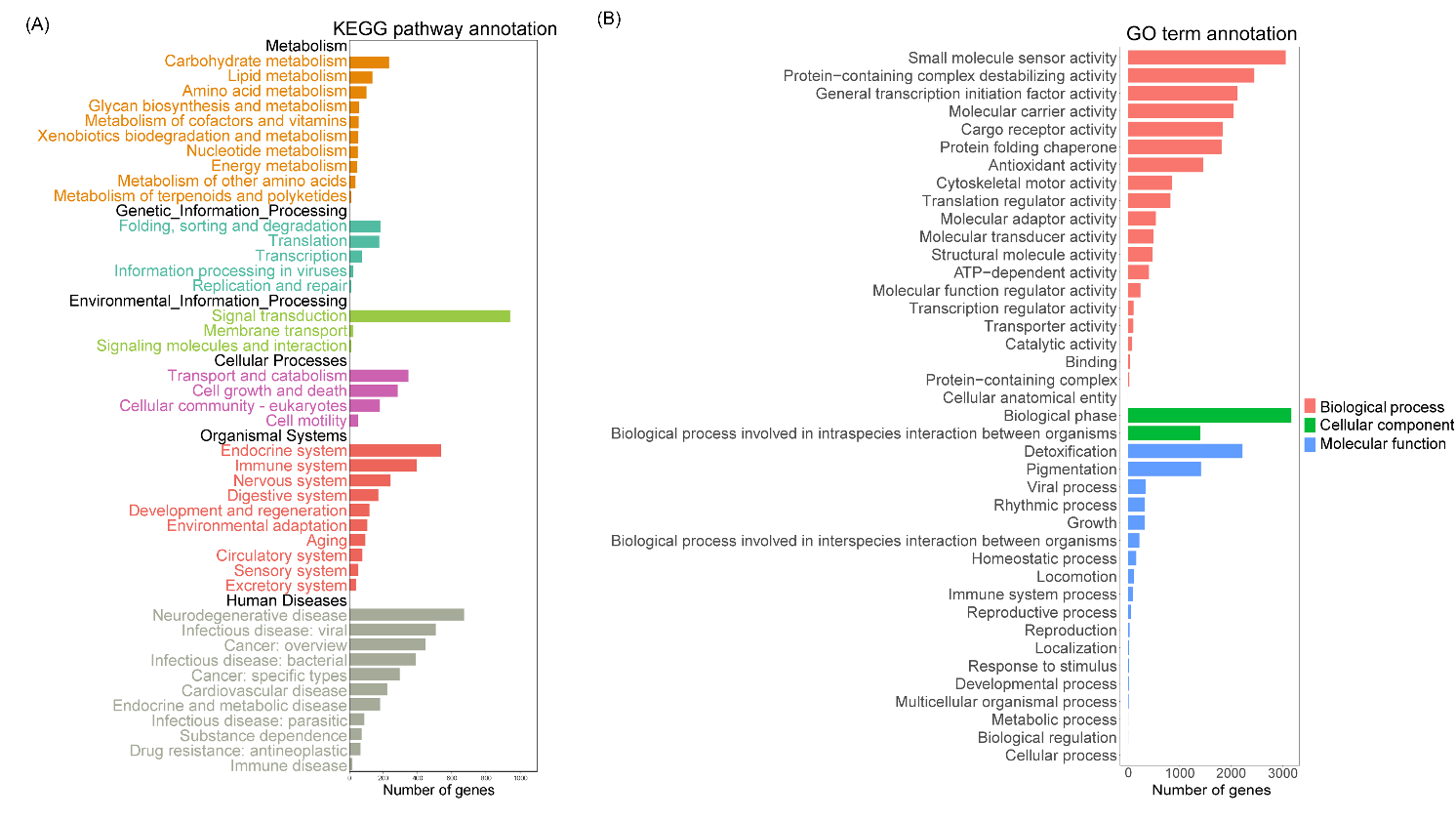


**Supplementary Figure 2.** Functional annotation of the genes identified from *B.dorsalis* antennal full-length transcriptome. (A) KEGG annotation of the genes identified from *B.dorsalis* antennal full-length transcriptome. (B) GO annotation of the genes identified from *B.dorsalis* antennal full-length transcriptome.

**
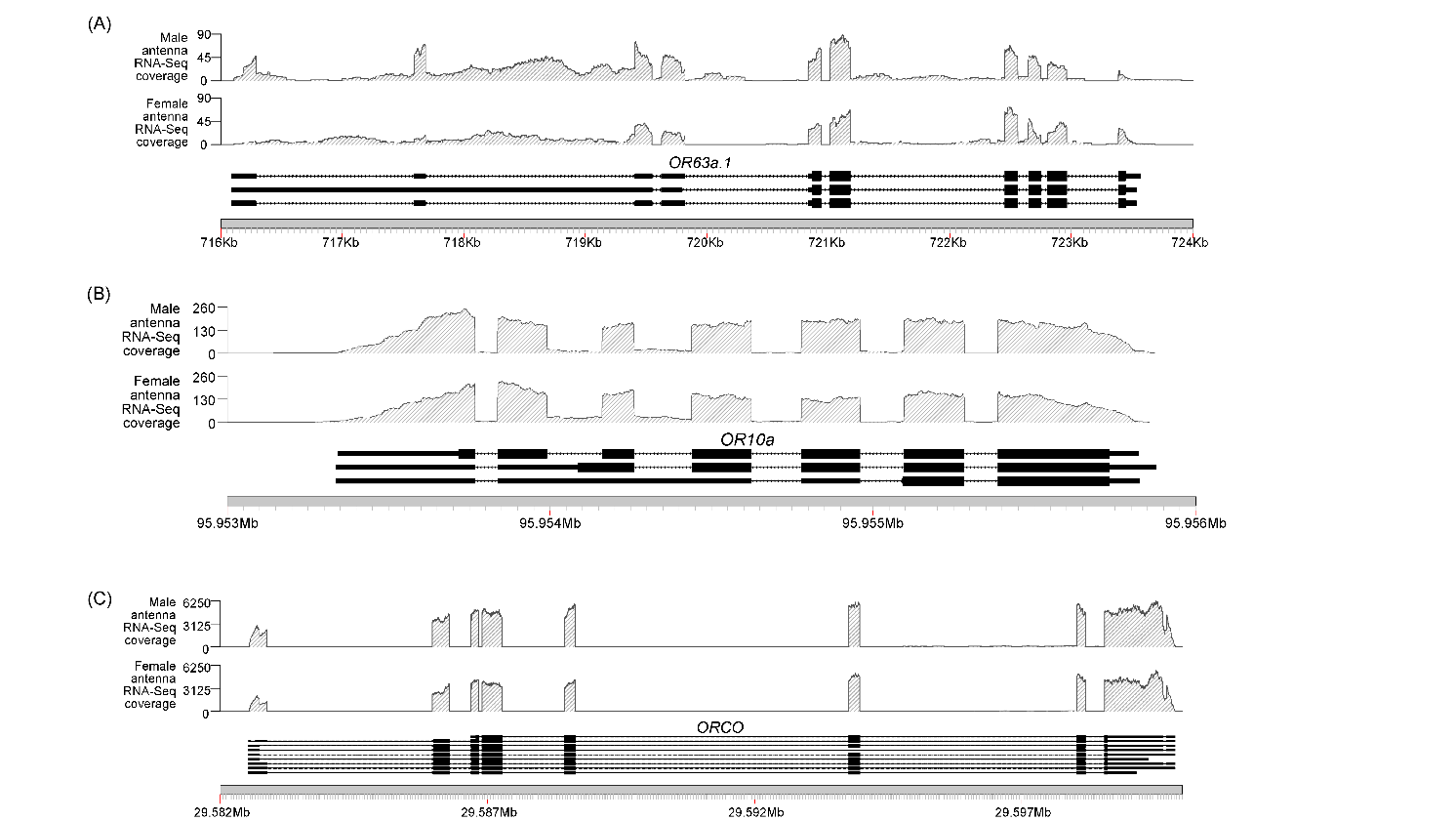
**

**Supplementary Figure 3.** The alternative splicing events of *B.dorsalis* odorant receptor genes identified from *B.dorsalis* antennal full-length transcriptome. (OR) genes and odorant co-receptor (ORCO) gene in *B.dorsalis* antennal full-length transcriptome. (A) The transcript structures of *BdorOR10a.* (B) The transcript structures of *BdorOR63a.1.* (C) The transcript structures of *BdorORCO.*

*
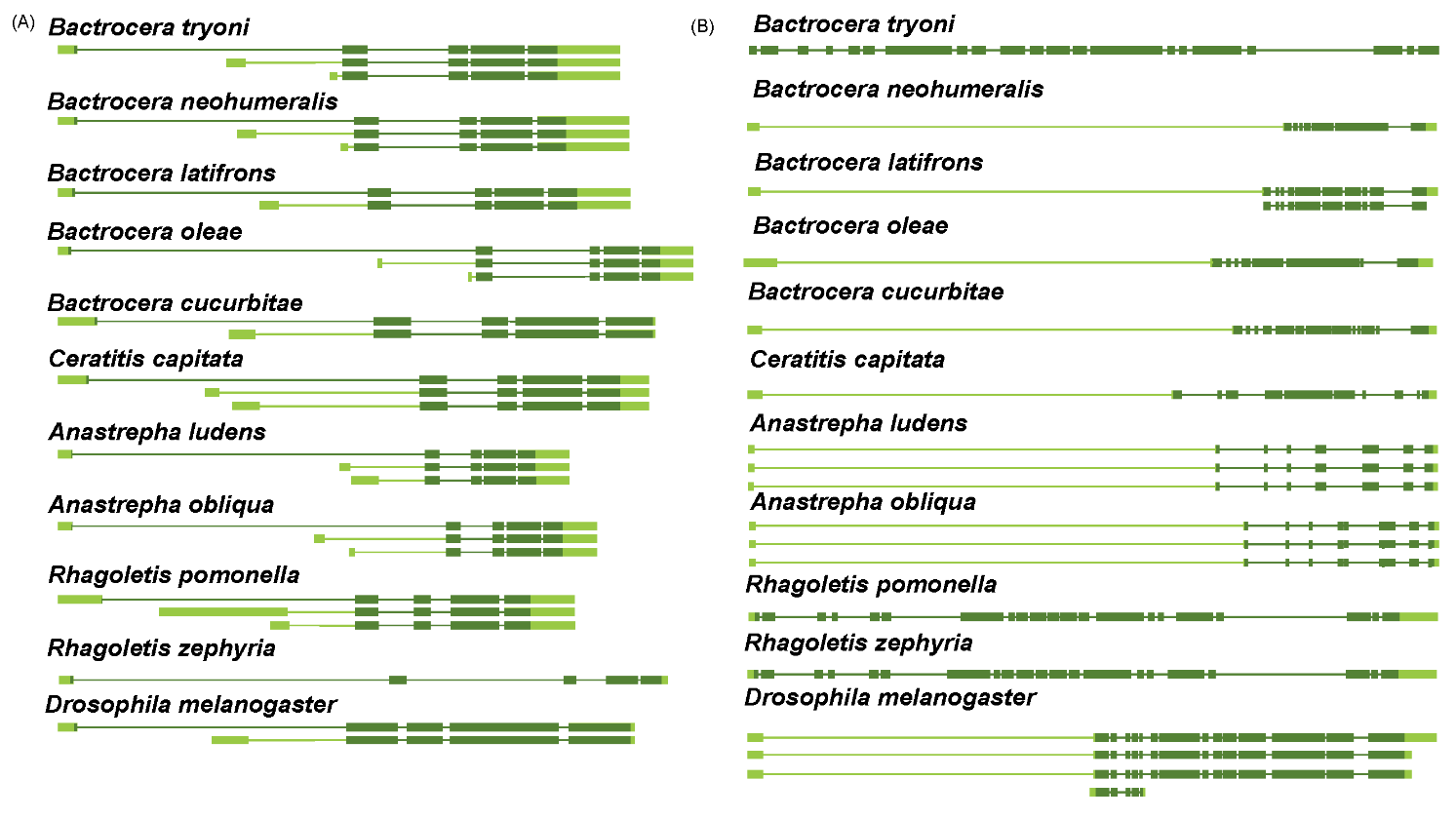
*

**Supplementary Figure 4.** The transcript structures of *ABCA13* and *CAT2* in *D.melanogaster* and 10 Tephritidae species. (A) The transcript structures of *CAT2* in *D.melanogaster* and 10 Tephritidae species. Dark green represents ORF region and light green represents UTR region. (B) The transcript structures of *ABCA13* in *D.melanogaster* and 10 Tephritidae species. Dark green represents ORF region and light green represents UTR region.


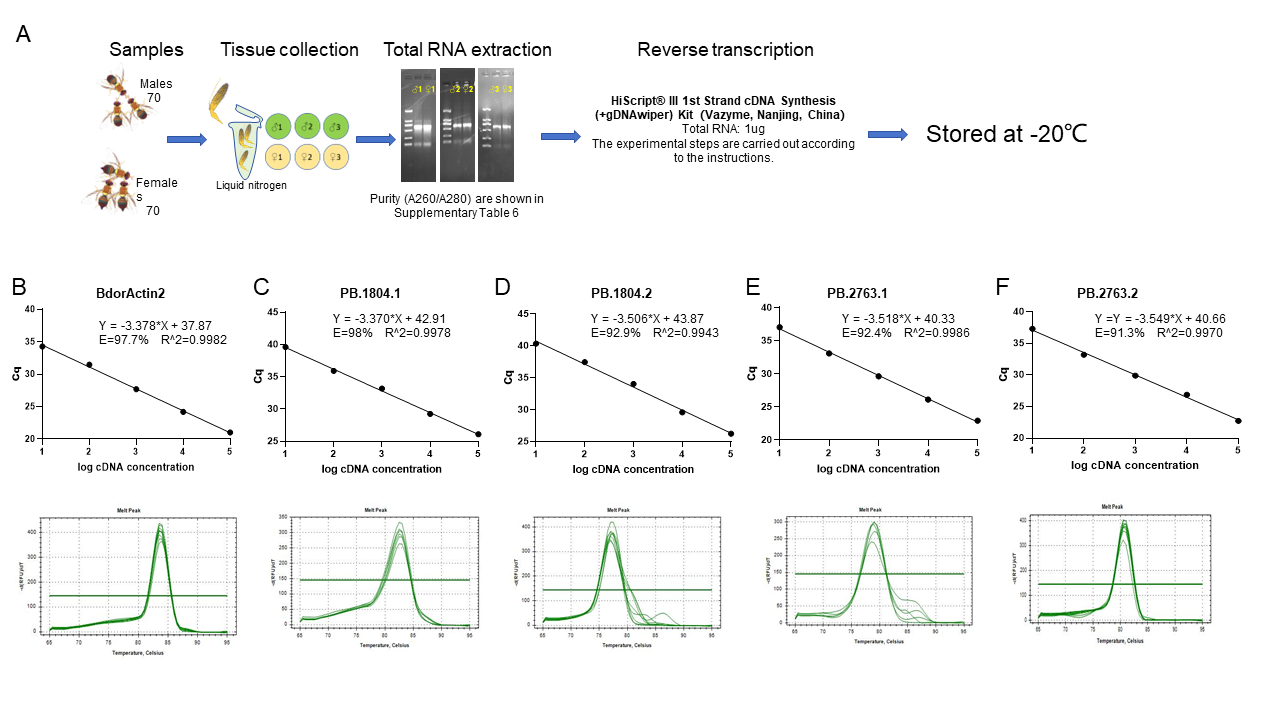


**Supplementary Figure 5.** RNA preparation process, qPCR primers amplification efficiency and specificity detection. (A) RNA preparation process. The gel image and the OD260/OD280 ratio indicate that the quality of RNA extraction meets the experimental requirements. The OD260/OD280 ratio are shown in Supplementary Table 6 (B) Amplification efficiency and amplification specificity of qPCR primers for BdorActin2. (C) Amplification efficiency and amplification specificity of qPCR primers for PB.1804.1. (D) Amplification efficiency and amplification specificity of qPCR primers for PB.1804.2. (E) Amplification efficiency and amplification specificity of qPCR primers for PB.2763.1. (F) Amplification efficiency and amplification specificity of qPCR primers for PB.2763.2.


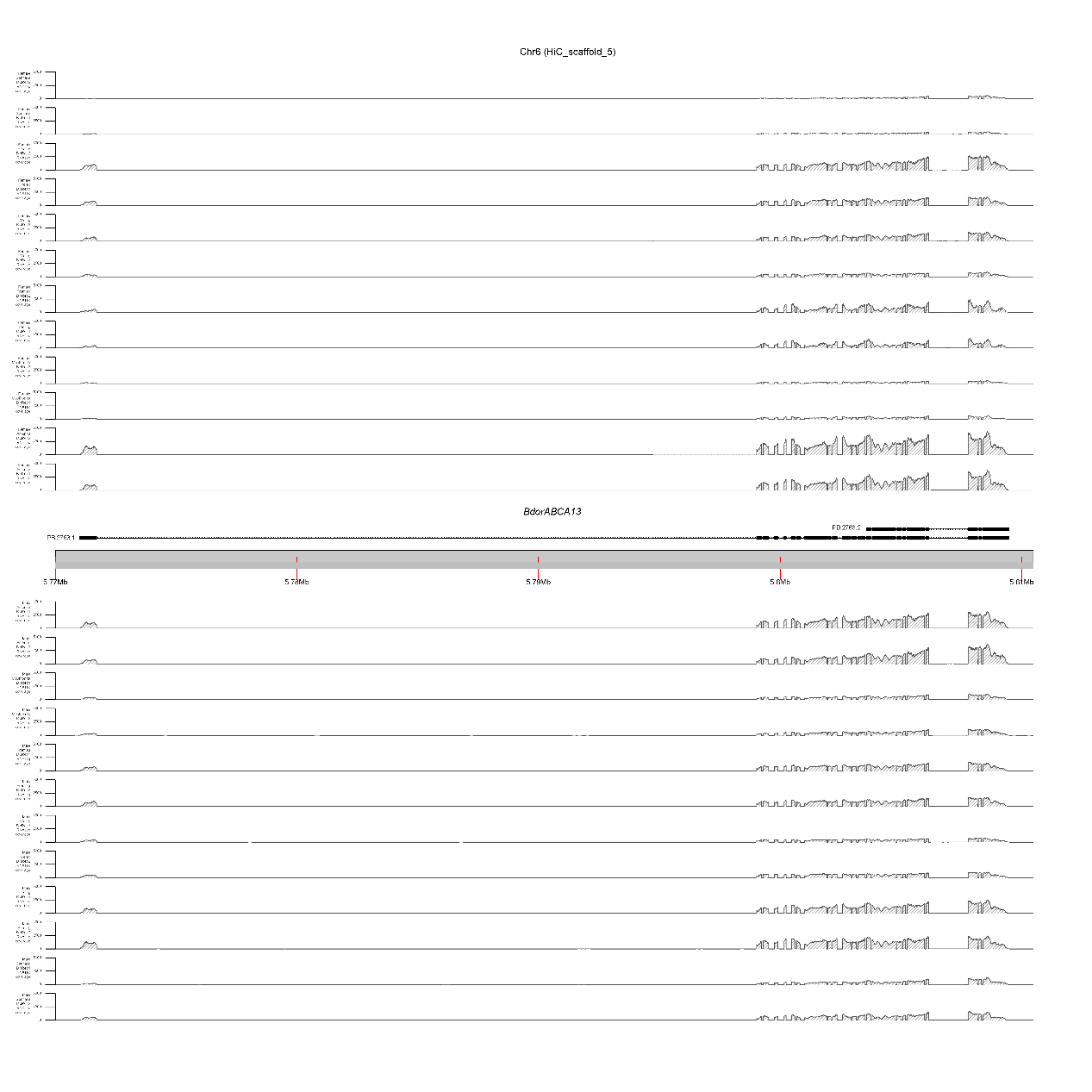


**Supplementary Figure 6.** RNA-Seq read depth of the peripheral nervous tissues of *Bactrocera dorsalis* associated with *BdorABCA13*.


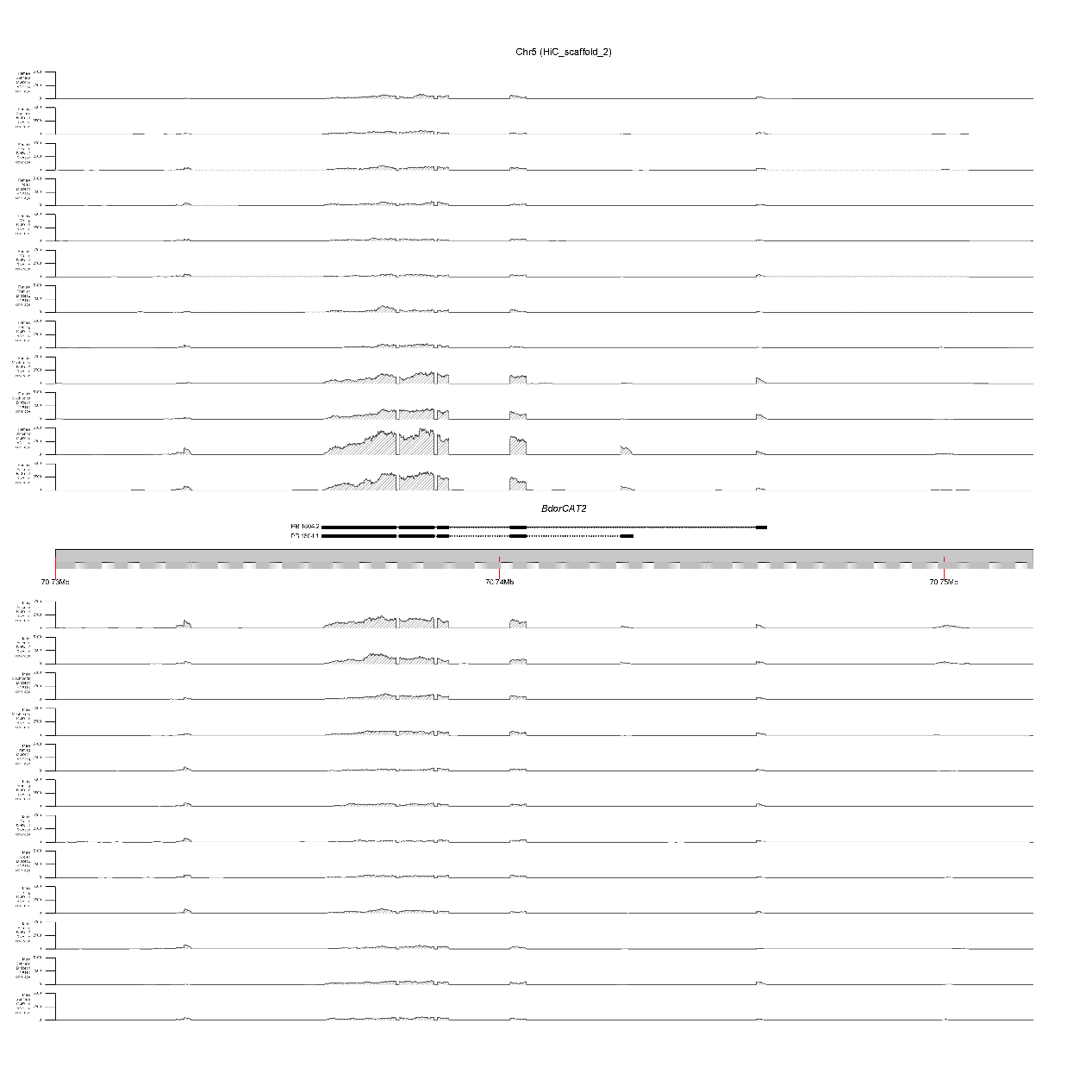


**Supplementary Figure 7.** RNA-Seq read depth of the peripheral nervous tissues of *Bactrocera dorsalis* associated with *BdorCAT2*.
